# Emergence of *Vibrio cholerae* O1 Sequence Type 75, South Africa, 2018–2020

**DOI:** 10.3201/eid2711.211144

**Published:** 2021-11

**Authors:** Anthony M. Smith, François-Xavier Weill, Elisabeth Njamkepo, Hlengiwe M. Ngomane, Ntsieni Ramalwa, Phuti Sekwadi, Juno Thomas

**Affiliations:** National Institute for Communicable Diseases, Johannesburg, South Africa (A.M. Smith, H.M. Ngomane, N. Ramalwa, P. Sekwadi, J. Thomas);; University of Pretoria, Pretoria, South Africa (A.M. Smith, N. Ramalwa);; Institut Pasteur, Paris, France (F.-X. Weill, E. Njamkepo)

**Keywords:** Vibrio cholerae, O1, ST75, sequence type 75, bacteria, antimicrobial resistance, enteric infections, South Africa

## Abstract

We describe the molecular epidemiology of cholera in South Africa during 2018–2020. *Vibrio cholerae* O1 sequence type (ST) 75 recently emerged and became more prevalent than the *V. cholerae* O1 biotype El Tor pandemic clone. ST75 isolates were found across large spatial and temporal distances, suggesting local ST75 spread.

The seventh cholera pandemic, caused by *Vibrio cholerae* O1 biotype El Tor (7PET), arrived in Africa during 1970 and became endemic in many countries on the continent (*1*). Cholera was first reported in South Africa in 1974 (*2*). However, South Africa is not considered a cholera-endemic area; outbreaks typically are associated with importation, particularly from neighboring countries. The last cholera outbreak in South Africa was triggered by imported cases from an outbreak in Zimbabwe during 2008; South Africa reported 12,706 cases during November 2008–April 2009 (*3*).

Globally, 7PET isolates are genetically homogeneous and linked to the Bay of Bengal in South Asia (*4*,*5*). Most 7PET isolates are multidrug-resistant sequence type (ST) 69 (*6*). Rarely, 7PET has a single-locus variant, ST515, in isolates from Africa belonging to lineage T10 (*7*). As of September 2021, all cholera isolates from South Africa have been characterized as 7PET ST69 by multilocus sequence typing (MLST).

South Africa actively surveils for cholera. Since the 2008–2009 outbreak, few cases have been identified: 5 during 2010–2014, most of which were imported, and none during 2015–2017. During 2008–2009, large outbreaks occurred in 3 provinces, Mpumalanga, Limpopo, and KwaZulu-Natal (*3*), but all were caused by imported cases from neighboring Zimbabwe and Mozambique. Therefore, given their experience, healthcare workers and laboratorians in these provinces typically will test for cholera in all cases of acute watery diarrhea.

In South Africa, the National Institute for Communicable Diseases (NICD) is notified of suspected cholera cases. NICD’s Centre for Enteric Diseases supports case investigations and receives all human and environmental *V. cholerae* isolates for further investigation. The case definition for confirmed cholera is isolation of *V. cholerae* O1 or O139 from a person with diarrhea. We investigated the molecular epidemiology of *V. cholerae* in South Africa during 2018–2020.

## The Study

During February 2018–January 2020, NICD received 102 *V. cholerae* isolates for testing; 9 were identified as *V. cholerae* O1. We characterized the bacteria by whole-genome sequencing, comparative genomics, and phylogenetic analysis (Appendix 1). The Human Research Ethics Committee of the University of the Witwatersrand (Johannesburg, South Africa) provided ethics approval for this study (protocol no. M160667).

Of 9 *V. cholerae* O1 isolates tested, we identified 2 ST69 (7PET) and 7 ST75 isolates. The ST69 isolates were collected in October 2018 from 2 cholera patients in a family cluster. The index case-patient had traveled to Zimbabwe, where an outbreak was ongoing (*8*), within the 7-day cholera incubation period before symptom onset. We confirmed these ST69 isolates belonged to the previously described highly antimicrobial-resistant Zimbabwe outbreak strain (*8*). The 7 ST75 isolates originated from KwaZulu-Natal and Limpopo Provinces. Five isolates were collected from patients with cholera, all adults 37–57 years of age; 2 isolates were from environmental samples collected during case investigations, 1 from sewage in Limpopo Province and 1 from river water in KwaZulu-Natal Province (Table 1). The 3 KwaZulu-Natal cases occurred ≈200–600 km apart; the first occurred in February 2018 and the last in January 2020. The 2 Limpopo cases occurred ≈70 km apart in the same district during November 2018. The Limpopo cases were >900 km from the KwaZulu-Natal cases. Epidemiologic investigations involved interviewing case-patients by using a standard case investigation form; visiting case-patients’ residences to inspect water and sanitation services and interview other household members; collecting stool samples from household members; and collecting environmental samples when indicated. Investigators found no evidence of importation from another country, epidemiologic links between cases, or secondary transmission.

**Table 1:** Clinical and demographic characteristics of 5 patients hospitalized with *Vibrio cholerae* O1 ST75 diagnosed from stool cultures and risk factors for *V. cholerae* infection, South Africa, 2018–2020*

Isolate no.	Province	Sample collection date	Patient age, y/sex	Clinical manifestations	Source of drinking water	Sanitation	Linked environmental samples	Type of environmental sample, isolate no.
YA00085869	KwaZulu-Natal	2018 Feb 8	37/F	Acute watery diarrhea, dehydration	Untreated river water	NA	N	NA
YA00132994	Limpopo	2018 Nov 9	38/M	Acute watery diarrhea, vomiting, dehydration	Untreated borehole water	Pit latrine and open defecation	N	NA
YA00134463	Limpopo	2018 Nov 20	45/M	Acute watery diarrhea, dehydration	Untreated borehole water	Flush toilets	Y	Sewage, OA01603367
YA00192016	KwaZulu-Natal	2019 Dec 29	49/M	Acute watery diarrhea, abdominal cramps, dehydration	Untreated river water	Pit latrine	Y	River water, CF00214281
YA00193061	KwaZulu-Natal	2020 Jan 12	57/F	Acute watery diarrhea, dehydration	NA	NA	N	NA

*All cases were diagnosed from stool cultures. All patients survived. NA, Not available; ST, sequence type. †Environmental samples tested positive for *V. cholerae *O1 ST75.

The 7 ST75 isolates showed notable features (Table 2). In particular, all carried the cholera toxin (CTX) prophage resembling CTX-2 with *ctxB1* genotype; *Vibrio* pathogenicity island 1 (VPI-1) encoding the toxin co-regulated pilus; and a variant form of *Vibrio* pathogenicity island 2 (VPI-2). However, isolates did not contain *Vibrio* seventh pandemic island I (VSP-I) and VSP-II. We noted several genomic islands (GIs), including VC-GI 119, but GI-05 was not present (Appendix 2). 

**Table 2:** Features of *Vibrio cholerae* O1 ST75 isolates, South Africa, 2018–2020*

Strain no.	Serotype	Biotype	AMR phenotype	AMR gene	Plasmids	*ctxB* allele	*tcpA*	*wbeT* mutation†	Lineage‡
YA00085869	Ogawa	El Tor	Pansusceptible	*qnrVC4*	None	*ctxB1*	*tcpA* ^N16961^	WT	L3b.1
YA00132994	Inaba	El Tor	Pansusceptible	*qnrVC4*	None	*ctxB1*	*tcpA* ^N16961^	B08	L3b.1
YA00134463	Inaba	El Tor	Pansusceptible	*qnrVC4*	None	*ctxB1*	*tcpA* ^N16961^	B08	L3b.1
OA01603367	Inaba	El Tor	Pansusceptible	*qnrVC4*	None	*ctxB1*	*tcpA* ^N16961^	B08	L3b.1
YA00192016	Ogawa	El Tor	Pansusceptible	*qnrVC4*	None	*ctxB1*	*tcpA* ^N16961^	WT	L3b.1
CF00214281	Ogawa	El Tor	Pansusceptible	*qnrVC4*	None	*ctxB1*	*tcpA* ^N16961^	WT	L3b.1
YA00193061	Ogawa	El Tor	Pansusceptible	*qnrVC4*	None	*ctxB1*	*tcpA* ^N16961^	WT	L3b.1

*AMR, antimicrobial resistance; ST, sequence type; WT, wild-type. †Nomenclature according to F.-X. Weill et al. (*5*). ‡Nomenclature according to H. Wang et al. (*9*).

The only antimicrobial-resistance determinant found in all ST75 isolates was the *qnrVC4* gene, located in the chromosomal superintegron. Various *qnrVC* alleles previously have been reported in the *Vibrionaceae* family and sometimes are associated with fluoroquinolone resistance (*10*,*11*). However, all ST75 isolates we analyzed showed fluoroquinolone susceptibility, MIC of ciprofloxacin 0.06 µg/mL, and susceptibility to all other tested antimicrobial drugs. This pansusceptibility sharply contrasts antimicrobial resistance trends observed in 7PET isolates from Africa, which reportedly became increasingly antimicrobial resistant over time; after the 2000s, none were susceptible to antimicrobial agents (*5*).

We further compared the ST75 isolates from South Africa with a larger global collection of 144 ST75, or closely related ST169, ST170, and ST182, genomes (Appendix 2), and constructed a maximum-likelihood phylogeny by using 49,540 SNPs (Figure). Our phylogenetic analysis showed that the 7 isolates from South Africa clustered in the L3b.1 clade, defined by H. Wang et al. (*9*), with a maximum pairwise distance of 22 SNPs. Isolates from Limpopo Province had a maximum pairwise distance of 1–6, but KwaZulu-Natal Province isolates had no SNP differences. Core-genome MLST showed Limpopo Province isolates differed from the KwaZulu-Natal Province isolates by 4–5 alleles (Appendix 1 Figure). The closest related isolates were collected in Russia from Rostov Oblast in 2005 and Republic of Kalmykia in 2011 and from Turkmenistan in Central Asia in 1965, but none of those isolates contained the CTX prophage. L3b.1 isolates from Taiwan containing the CTX prophage *ctxB3* allele were more distant.

**Figure:**
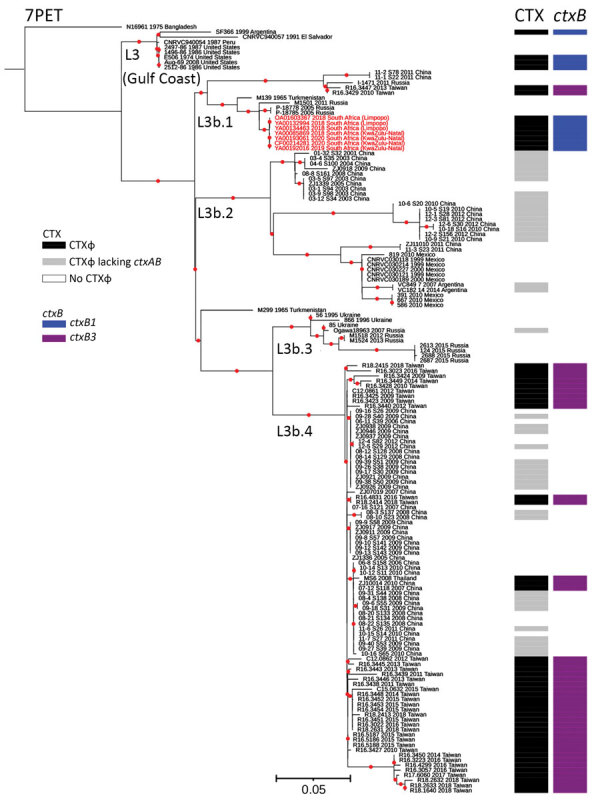
Maximum-likelihood phylogenomic tree for *Vibrio cholerae* O1 sequence type (ST) 75 isolates collected from South Africa, 2018–2020. The tree represents phylogeny for 7 *V. cholerae* O1 ST75 isolates from South Africa (red text); 144 sequences from a global collection of ST75, or closely related ST169, ST170, and ST182 isolates; and 1 7PET *V. cholerae* O1 sequence. The 7PET genome N16961 (ST69) was used as an outgroup. For each genome, its name; year of collection, when known; and country of isolation, plus province of isolation for isolate from South Africa, are shown at the tips of the tree. The lineages, presence of CTXɸ prophage or its variant form, and types of *ctxB* alleles are also shown. The 7PET outgroup genome, N16961, contains CTXɸ with a *ctxB3* allele (not represented in the figure). Red dots indicate bootstrap values >95%. Scale bar indicates the number of nucleotide substitutions per variable site. 7PET, seventh pandemic *V. cholerae* O1 El Tor; CTXɸ, cholera toxin phi prophage; *ctxB*, cholera toxin B subunit gene.

Emergence of ST75 L3b.1 clade in South Africa is cause for concern. Recent studies on *V. cholerae* O1 isolated in Taiwan (*12*) and China (*13*) reported emerging and potential toxigenic ST75. Genomic signatures of these ST75 isolates closely resembled the US Gulf Coast *V. cholerae* O1 clone that emerged in 1973 (*14*). In particular, an investigation of *V. cholerae* O1 isolated during 2002–2018 in Taiwan showed that ST75 emerged there in 2009 and now is more prevalent than the ST69 pandemic clone (*12*). Our findings from South Africa align with the findings from Taiwan, showing that ST75 isolates outnumber ST69 isolates. 

One limitation of our study is that we used reference laboratory data and a review of published *V. cholerae* O1 data to conclude that all previous cholera isolates in South Africa characterized by MLST were *V. cholerae* O1 biotype El Tor ST69. However, we cannot exclude the possibility that *V. cholerae* O1 isolates not characterized by MLST, particularly those from environmental samples, could have been non-ST69.

Epidemic 7PET lineage cholera demands an aggressive public health response to prevent outbreaks. In contrast, sporadic *V. cholerae* O1 infections mediated by other lineages, including those carrying toxin co-regulated pilus and CTX genes, typically are not epidemic-prone; most are associated with sporadic cases that rarely lead to secondary transmission (*15*). Tailoring the public health response to the degree of epidemic risk would be invaluable, especially in resource-limited settings. In countries that are not cholera-endemic but are at high risk for cholera introductions, conventional laboratory determination of *V. cholerae* O1, even complemented by identifying *ctxA* or *ctxB* genes, might be insufficient. Typing resolution of genomics, which distinguishes between 7PET and nonepidemic lineages, can elucidate the local and global epidemiology of cholera and inform public health decisions.

## Conclusions

The emergence and dominance of nonepidemic, non-7PET, *V. cholerae* ST75 L3b.1 in South Africa requires close monitoring. The spatiotemporal pattern suggests local spread, possibly indicating a geographically widespread risk for sporadic disease from this strain. South Africa should strengthen its disease and environmental surveillance systems to identify non-pandemic ST75 strains, define local epidemiology, and inform an appropriate public health response.

Appendix 1 Additional information on methods used to characterize emergence of *Vibrio cholerae* O1 Sequence Type 75 collected from South Africa during 2018–2020.

Appendix 2 Genomic sequences used to study of emergence of Vibrio cholerae O1 Sequence Type 75 collected from South Africa during 2018–2020.
