# Supplementary material for: Emergence of Vibrio cholerae O1 Sequence Type 75, South Africa, 2018–2020
Source: Emerg Infect Dis. 2021 Nov;27(11):2927–31. doi: 10.3201/eid2711.211144 (PMC8544974; doi:10.3201/eid2711.211144)
Supplement: Appendix 1 — Additional information on methods used to characterize emergence of Vibrio cholerae O1 Sequence Type 75 collected from South Africa during 2018–2020. [file 21-1144-Techapp-s1.pdf]

Article DOI: https://doi.org/10.3201/eid2711.211144

Emergence of *Vibrio cholerae* O1 Sequence Type 75, South Africa, 2018–2020

Appendix 1

Materials and Methods

Phenotypic Characterization

We confirmed the identification of *Vibrio cholerae* isolates by using standard phenotypic microbiological identification and serotyping techniques. In brief, we subcultured bacteria onto 5% blood agar (National Health Laboratory Service, Diagnostic Media Products, https://www.nhls.ac.za) and thiosulfate citrate bile salts sucrose agar (Diagnostic Media Products) to check for purity of cultures. We identified bacteria by using the VITEK-2 COMPACT 15 (bioMérieux, https://www.biomerieux.com) automated microbial identification system. We determined serogrouping and serotyping by the slide agglutination method with polyvalent antisera and monospecific Inaba and Ogawa antisera (Mast Group Ltd, https://www.mast-group.com).

We performed antimicrobial susceptibility testing as follows. We determined the MIC of ampicillin, amoxicillin/clavulanate, cefepime, trimethoprim/sulfamethoxazole, chloramphenicol, ciprofloxacin, tetracycline, kanamycin, streptomycin, imipenem, and azithromycin by using the ETEST method (bioMérieux). We used the Clinical and Laboratory Standards Institute (CLSI) interpretative criteria for antimicrobial susceptibility testing of *Vibrio* species (M45) (*1*), when available. For antimicrobial drugs not listed on this *Vibrio* species in M45, we used the CLSI interpretative criteria for *Enterobacteriaceae*/*Salmonella* species (M100) (*2*). Interpretive criteria for streptomycin are not available in either CLSI document; thus, we used susceptibility $\leq$16 µg/mL and resistance $\geq$32 µg/mL.

PCR for Toxin Detection and Biotyping

We used real-time PCR to detect cholera toxin, *ctxA* gene (*3*). We used conventional PCR and analyzed PCR products using agarose gel electrophoresis to detect the presence of allelic variants of the toxin co-regulated pilus, *tcpA* gene, which determined the biotype as classical or El Tor type of *V. cholerae* O1 (*4*).

Genomic DNA Isolation from Bacteria and Whole-Genome Sequencing

We isolated genomic DNA from bacteria by using an Invitrogen PureLink Microbiome DNA Purification Kit (ThermoFisher Scientific, https://www.thermofisher.com). We performed whole-genome sequencing (WGS) by using NextSeq (Illumina, https://www.illumina.com) next-generation sequencing technology, and prepared DNA libraries by using a Nextera DNA Flex Library Preparation Kit (Illumina), before running 2 × 150 bp paired-end sequencing runs with ≈80 times coverage.

Additional Genomic Data

We downloaded and included raw sequence files from 71 *V. cholerae* genomes described by D. Domman et al. (*5*), A.R. Reimer et al. (*6*), Y. Luo et al. (*7*), S.S. Watve et al. (*8*), and Y.H. Tu et al. (*9*) from the European Nucleotide Archive (https://www.ebi.ac.uk/ena/browser/home) in the study (Appendix 2, https://wwwnc.cdc.gov/eid/article/27/11/21-1144-App2.xlxs). We downloaded and included 74 unpublished, complete, or assembled genomes described by J.F. Heidelberg et al. (*10*), K. Okada et al. (*11*), D. Hu et al. (*12*), K.V. Kuleshov et al. (*13*), H. Wang et al. (*14*) from GenBank in this study (Appendix 2).

Genomic Sequence Analyses

We used FqCleanER version 3.0 (https://gitlab.pasteur.fr/GIPhy/fqCleanER) to eliminate adaptor sequences (*15*), correct sequencing errors (*16*), and discard low-quality short-reads. We generated assemblies by using SPAdes version 3.15.0 (*17*).

We performed in silico multilocus sequence typing (MLST) on assembled sequences for the entire *V. cholerae* dataset by using MLST version 2.0 (https://cge.cbs.dtu.dk/services/MLST/) (*18*). We analyzed various genetic markers by using BLAST version 2.2.26 (https://blast.ncbi.nlm.nih.gov) against reference sequences of the CTX prophage (CTX-1, GenBank accession no. AE003852, coordinates 1566967–1573281; CTX-2, GenBank accession no. CP001486, coordinates 852233–858550), the *ctxB* gene (GenBank accession no. AE003852, coordinates 1566967–1567341), the toxin co-regulated pilus (TCP) genes (GenBank accession no. AE003852, coordinates 890449–891123), *Vibrio* pathogenicity island (VPI) 1 (VPI-1, GenBank accession no. AE003852, coordinates 873242–914124), VPI-2 (GenBank accession no. AE003852, coordinates 1896092- 1952861), *Vibrio* seventh pandemic island (VSP) I (VSP-I, GenBank accession no. AE003852, coordinates 175343–189380), and VSP-II (GenBank accession no. AE003852, coordinates 523156–550021). We used VCGIDB (http://leb.snu.ac.kr/vcgidb/index) to determine the presence and type of genomic islands and ResFinder v4.0.1 (https://cge.cbs.dtu.dk/services/ResFinder) to determine acquired antimicrobial resistance genes.

Phylogenetic Analysis

We performed phylogenetic analysis on the 7 *V. cholerae* O1 ST75 isolates from this study and 145 additional genomes. We mapped the paired-end reads and genome assemblies onto the reference genome of *V. cholerae* O1 El Tor N16961, also known as A19 (GenBank accession nos. LT907989 and LT907990) by using Snippy version 4.1.0 and BWA version 0.7.17 (https://github.com/tseemann/snippy). We called SNPs by using Snippy version 4.6.0 and Freebayes version 1.3.2 (https://github.com/tseemann/snippy) under the following constraints: mapping quality of 60, minimum base quality of 13, minimum read coverage of 4, and a 75% read concordance at a locus for a variant to be reported. We aligned core genome SNPs in Snippy version 4.1.0 for phylogeny inference. We masked repetitive regions, such as insertion sequences and the TLC-RS1-CTX region, and recombinogenic VSP-II regions in the alignment (*19*). We built a maximum-likelihood phylogenetic tree from 49,540 aligned chromosomal SNPs, by using RAxML version 8.2.12, under the general time-reversible model with 200 bootstraps (*20*). The final tree was rooted on the N16961 genome, which we visualized by using Interactive Tree of Life (iTOL) version 6 (https://itol.embl.de) (*21*).

Core-Genome Multilocus Sequence Typing

We uploaded and investigated raw genomic sequencing data from FastQ files of paired-end reads to the EnteroBase web-based platform (http://enterobase.warwick.ac.uk/species/index/vibrio). We used the EnteroBase core-genome multilocus sequence typing (cgMLST) tool to perform a genomic comparison of isolates. The cgMLST scheme (Vibrio cgMLST + HierCC) incorporates 1,128 core genes. We displayed phylogenetic cluster analysis of cgMLST data by using a GrapeTree-generated minimum spanning tree with the MSTree V2 algorithm (https://bitbucket.org/enterobase/enterobase-web/wiki/GrapeTree) (*22*). We deposited short-read sequence data in the NCBI (https://www.ncbi.nlm.nih.gov) Sequence Read Archive under BioProject identification no. PRJEB39740.

All isolates showed $\leq$4 allele differences when compared against each other, indicative of highly genetically related isolates. We used the cgMLST hierarchical cluster tool of EnteroBase to further interrogate the isolates. At hierarchical cluster level 5 (HC5), where isolates are clustered at 5 allele differences, all isolates grouped into a single HC5:1000. At HC2, the isolates are split into 2 clusters: HC2:2146, which included isolates recovered in KwaZulu-Natal Province; and HC2:1003, which included isolates recovered in Limpopo Province. This finding suggests that the isolates from KwaZulu-Natal Province are slightly genetically different to those from Limpopo Province.

References

1. Clinical and Laboratory Standards Institute. Methods for antimicrobial dilution and disk susceptibility testing of infrequently isolated or fastidious bacteria; approved guideline, third edition M45). The Institute: Wayne (PA); 2015.

2. Clinical and Laboratory Standards Institute. Performance standards for antimicrobial susceptibility testing; twenty-seventh informational supplement (M100–S27). The Institute: Wayne (PA): 2017.

3. Koskela KA, Matero P, Blatny JM, Fykse EM, Olsen JS, Nuotio LO, et al. A multiplatform real-time polymerase chain reaction detection assay for *Vibrio cholerae*. Diagn Microbiol Infect Dis. 2009;65:339–44. PubMed https://doi.org/10.1016/j.diagmicrobio.2009.07.009

4. Keasler SP, Hall RH. Detecting and biotyping *Vibrio cholerae* O1 with multiplex polymerase chain reaction. Lancet. 1993;341:1661. PubMed https://doi.org/10.1016/0140-6736(93)90792-F

5. Domman D, Quilici ML, Dorman MJ, Njamkepo E, Mutreja A, Mather AE, et al. Integrated view of *Vibrio cholerae* in the Americas. Science. 2017;358:789–93. PubMed https://doi.org/10.1126/science.aao2136

6. Reimer AR, Van Domselaar G, Stroika S, Walker M, Kent H, Tarr C, et al.; V. cholerae Outbreak Genomics Task Force. Comparative genomics of *Vibrio cholerae* from Haiti, Asia, and Africa. Emerg Infect Dis. 2011;17:2113–21. PubMed https://doi.org/10.3201/eid1711.110794

7. Luo Y, Octavia S, Jin D, Ye J, Miao Z, Jiang T, et al. US Gulf–like toxigenic O1 *Vibrio cholerae* causing sporadic cholera outbreaks in China. J Infect. 2016;72:564–72. PubMed https://doi.org/10.1016/j.jinf.2016.02.005

8. Watve SS, Chande AT, Rishishwar L, Mariño-Ramírez L, Jordan IK, Hammer BK. Whole-genome sequences of 26 *Vibrio cholerae* isolates. Genome Announc. 2016;4:e01396. PubMed https://doi.org/10.1128/genomeA.01396-16

9. Tu YH, Chen BH, Hong YP, Liao YS, Chen YS, Liu YY, et al. Emergence of *Vibrio cholerae* O1 sequence type 75 in Taiwan. Emerg Infect Dis. 2020;26:164–6. PubMed https://doi.org/10.3201/eid2601.190934

10. Heidelberg JF, Eisen JA, Nelson WC, Clayton RA, Gwinn ML, Dodson RJ, et al. DNA sequence of both chromosomes of the cholera pathogen *Vibrio cholerae*. Nature. 2000;406:477–83. PubMed https://doi.org/10.1038/35020000

11. Okada K, Na-Ubol M, Natakuathung W, Roobthaisong A, Maruyama F, Nakagawa I, et al. Comparative genomic characterization of a Thailand-Myanmar isolate, MS6, of *Vibrio cholerae* O1 El Tor, which is phylogenetically related to a "US Gulf Coast" clone. PLoS One. 2014;9:e98120. PubMed https://doi.org/10.1371/journal.pone.0098120

12. Hu D, Liu B, Feng L, Ding P, Guo X, Wang M, et al. Origins of the current seventh cholera pandemic. Proc Natl Acad Sci U S A. 2016;113:E7730–9. PubMed https://doi.org/10.1073/pnas.1608732113

13. Kuleshov KV, Vodop’ianov SO, Markelov ML, Dedkov VG, Kermanov AV, Kruglikov VD, et al. Draft genome sequences of *Vibrio cholerae* O1 El Tor strains 2011EL-301 and P-18785, isolated in Russia. Genome Announc. 2013;1:e00659. PubMed https://doi.org/10.1128/genomeA.00659-13

14. Wang H, Yang C, Sun Z, Zheng W, Zhang W, Yu H, et al. Genomic epidemiology of *Vibrio cholerae* reveals the regional and global spread of two epidemic non-toxigenic lineages. PLoS Negl Trop Dis. 2020;14:e0008046. PubMed https://doi.org/10.1371/journal.pntd.0008046

15. Criscuolo A, Brisse S. AlienTrimmer: a tool to quickly and accurately trim off multiple short contaminant sequences from high-throughput sequencing reads. Genomics. 2013;102:500–6. PubMed https://doi.org/10.1016/j.ygeno.2013.07.011

16. Liu Y, Schröder J, Schmidt B. Musket: a multistage k-mer spectrum-based error corrector for Illumina sequence data. Bioinformatics. 2013;29:308–15. PubMed https://doi.org/10.1093/bioinformatics/bts690

17. Bankevich A, Nurk S, Antipov D, Gurevich AA, Dvorkin M, Kulikov AS, et al. SPAdes: a new genome assembly algorithm and its applications to single-cell sequencing. J Comput Biol. 2012;19:455–77. PubMed https://doi.org/10.1089/cmb.2012.0021

18. Larsen MV, Cosentino S, Rasmussen S, Friis C, Hasman H, Marvig RL, et al. Multilocus sequence typing of total-genome-sequenced bacteria. J Clin Microbiol. 2012;50:1355–61. PubMed https://doi.org/10.1128/JCM.06094-11

19. Weill FX, Domman D, Njamkepo E, Tarr C, Rauzier J, Fawal N, et al. Genomic history of the seventh pandemic of cholera in Africa. Science. 2017;358:785–9. PubMed https://doi.org/10.1126/science.aad5901

20. Stamatakis A. RAxML-VI-HPC: maximum likelihood–based phylogenetic analyses with thousands of taxa and mixed models. Bioinformatics. 2006;22:2688–90. PubMed https://doi.org/10.1093/bioinformatics/btl446

21. Letunic I, Bork P. Interactive Tree Of Life (iTOL) v5: an online tool for phylogenetic tree display and annotation. Nucleic Acids Res. 2021;49(W1):W293–6. PubMed https://doi.org/10.1093/nar/gkab301

22. Zhou Z, Alikhan NF, Sergeant MJ, Luhmann N, Vaz C, Francisco AP, et al. GrapeTree: visualization of core genomic relationships among 100,000 bacterial pathogens. Genome Res. 2018;28:1395–404. PubMed https://doi.org/10.1101/gr.232397.117

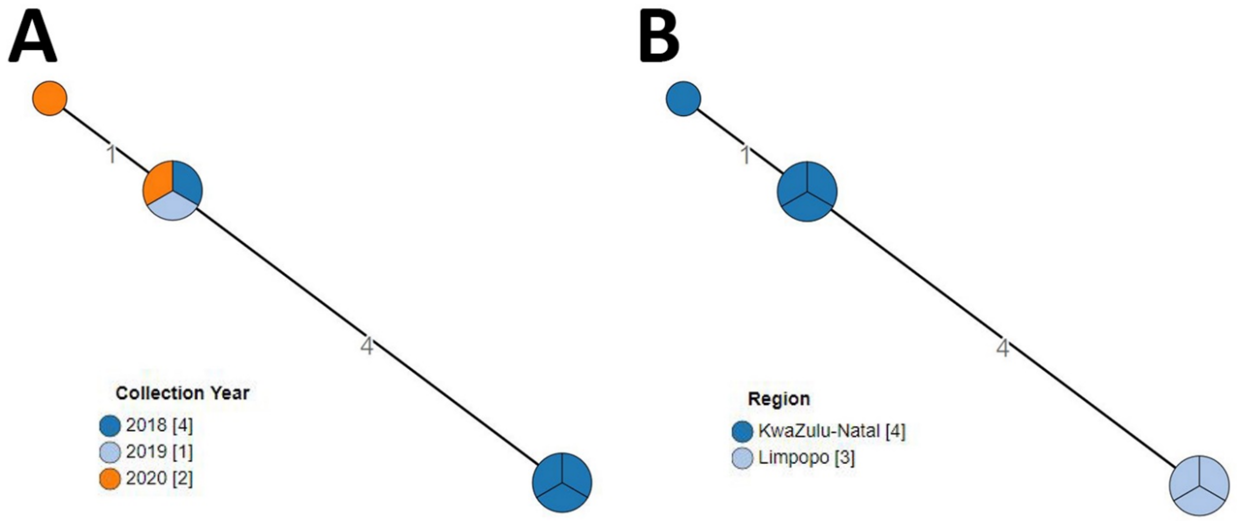

Appendix 1 Figure. Minimum spanning tree drawn by using cgMLST data from 7 *Vibrio cholerae* O1 sequence type 75 isolates collected in South Africa, 2018–2020. A) Isolate collection year; B) region of isolate collection. Circular nodes represent isolates having identical cgMLST profiles; the larger the node, the more isolates included. Number values between adjacent nodes indicate the number of allele differences between nodes. cgMLST, core-genome multilocus sequence typing.
